# Identification of microbial interaction network: zero-inflated latent Ising model based approach

**DOI:** 10.1186/s13040-020-00226-7

**Published:** 2020-10-07

**Authors:** Jie Zhou, Weston D. Viles, Boran Lu, Zhigang Li, Juliette C. Madan, Margaret R. Karagas, Jiang Gui, Anne G. Hoen

**Affiliations:** 1 Department of Biomedical Data Science, Geisel School of Medicine, Dartmouth College, Hanover, NH USA; 2 Department of Epidemiology, Geisel School of Medicine, Dartmouth College, Hanover, NH USA; 3 Department of Mathematics and Statistics, University of Southern Maine, Portland, ME USA; 4 Department of Biostatistics, University of Florida, Gainesville, FL USA

**Keywords:** Gut microbiota, Microbial interaction network, Latent Ising model, Dynamic programming, High-dimensional data, Sparse estimation

## Abstract

**Background:**

Throughout their lifespans, humans continually interact with the microbial world, including those organisms which live in and on the human body. Research in this domain has revealed the extensive links between the human-associated microbiota and health. In particular, the microbiota of the human gut plays essential roles in digestion, nutrient metabolism, immune maturation and homeostasis, neurological signaling, and endocrine regulation. Microbial interaction networks are frequently estimated from data and are an indispensable tool for representing and understanding the conditional correlation between the microbes. In this high-dimensional setting, zero-inflation and unit-sum constraint for relative abundance data pose challenges to the reliable estimation of microbial interaction networks.

**Methods and Results:**

To identify the microbial interaction network, the *zero-inflated latent Ising* (ZILI) model is proposed which assumes the distribution of relative abundance relies only on finite latent states and provides a novel way to solve issues induced by the unit-sum and zero-inflation constrains. A two-step algorithm is proposed for the model selection of ZILI. ZILI is evaluated through simulated data and subsequently applied to an infant gut microbiota dataset from New Hampshire Birth Cohort Study. The results are compared with results from Gaussian graphical model (GGM) and dichotomous Ising model (DIS). Providing ZILI is the true data-generating model, the simulation studies show that the two-step algorithm can identify the graphical structure effectively and is robust to a range of parameter settings. For the infant gut microbiota dataset, the final estimated networks from GGM and ZILI turn out to have significant overlap in which the ZILI tends to select the sparser network than those from GGM. From the shared subnetwork, a hub taxon Lachnospiraceae is identified whose involvement in human disease development has been discovered recently in literature.

**Conclusions:**

Constrains induced by relative abundance of microbiota such as zero inflation and unit sum render the conditional correlation analysis unreliable for conventional methods such as GGM. The proposed optimal categoricalization based ZILI model provides an alternative yet elegant way to deal with these difficulties. The results from ZILI have reasonable biological interpretation. This model can also be used to study the microbial interaction in other body parts.

## Introduction

The human microbiome, the collection of trillions of microbial organisms that live in our body spaces, belong to one of thousands of different species [1, 2]. The organisms that inhabit the human gut are an additional source of genetic diversity that can influence metabolism and modulate drug interactions [3]. Recent advances in genomic technologies enable production of thousands of 16S rRNA sequences per sample [4] and are powerful tools to explore the basic biology about human microbiome. Nevertheless, analyzing microbiome data and converting them into meaningful biological insights are still challenging tasks. First, the observed absolute abundance in sequencing experiment cannot inform the real absolute abundance of molecules in the sample which can be attributed to the sequence depth associated with the experiment. Multiple normalization methods have been proposed in literature to solve this problem among which total sum scaling (TSS) has been widely used in practice [5–9]. TSS scales each sample by the total read count and yields the relative abundance. However, the statistical analysis based on relative abundance can easily lead to spurious association due to the unit-sum constraint [10–14]. Further complicating the analysis of microbiome data is the zero-inflated distribution of read count [3]. As for the dataset in “Restults from the relative abundance of gut microbiota” section, among the 134 taxa, there are only 6 taxa for which the proportions of nonzero observations are greater than 80%. Zero inflation stems from the fact that the majority of the amplicon sequence variants (ASVs) either physically do not exist in the subject or are below the detection threshold for the given sample [2]. Another hurdle for analyzing the microbiome data is its high-dimensionality which usually involves hundreds of microbes; consequently, models equipped for this modeling task should be employed.

Microbial interaction network (MIN) is an indispensable tool for representing and understanding the relationships among the microbes [1, 15–17]. Traditionally, the interactions among the microbes are discovered through co-culture experiments which routinely involve only small number of species in an artificial community [18, 19]. Modern researches try to use the data from real environments such as human gut to infer the association among the microbes [20–23]. The corresponding statistical inferences of MIN based on these observational data have received much attention in recent years; however, the roadblocks mentioned above hinder the effective inference of MIN. As a compromise, most of the existing studies infer the MIN under the oversimplified assumptions [24, 25]. Especially, [24] ignores the unit-sum constraint and only considers the microbes for which the proportions of nonzero observation are higher than a given threshold; while in order to deal with the problem of zero inflation, [25] pools all the sparse taxa together and forms a composite taxon which is no longer sparse.

In light of the difficulties in MIN inference, in this paper we propose the zero-inflated latent Ising model (ZILI) for MIN aiming to address the roadblocks for analyzing the relative abundance of microbiome, i.e., (1) unit-sum constraint; (2) zero inflation; (3) high dimensionality. Latent models such as hidden Markov models [26], state space models [27] et al. have been widely used in economics, engineering and biology among many others. Despite their popularity across disciplines, latent models have not been investigated for microbiome data yet. Incidentally, [28] finds that the microbiota in human vagina could be characterized by finite states which provided a simple and intuitive understanding about the MIN in vagina. Inspired by the work in [28], in ZILI we assume that each of the *p* microbes in microbiota can be characterized by a latent discrete random variable *Z*_*j*_(1≤*j*≤*p*). While for the random vector **Z**=(*Z*_1_,⋯,*Z*_*p*_), the multiclass Ising model is employed to characterize the joint distribution of **Z**. The relative abundances for each microbe are assumed to come from a zero-inflated mixture distribution which depends on the realization of **Z**. Under this modeling framework, we propose a two-step algorithm for the model selection of ZILI. Specifically, in first step we estimate the states for each component of **Z** by transforming the relative abundances into categorical data. This step is implemented by an efficient dynamic programming algorithm. Based on the estimated state, in second step we use *L*_1_-penalized group logistic regression to select the nonzero parameters involved in ZILI. In this way, the difficult issues, such as the unti-sum constraint and zero inflation, will not be the concerns for the data analysis. However, the cost for such simplication is the possible information loss brought by the categorization of relative abundance. Through simulated data, we investigate the performance of two-step algorithm and demonstrate its superiority over traditional Gaussian graphical model (GGM) and dichotomous Ising models (DIS) given that ZILI is the underlying data-generating model. We then apply both ZILI and GGM to an infant gut microbiome dataset from the New Hampshire Birth Cohort Study. It turns out the networks estimated by ZILI and GGM share a statistically significant part and ZILI shows the tendency to select sparser network than GGM. Within the shared subnetwork, Lachnospiraceae is identified as the hub taxon. On the other hand, recent researches have found that Lachnospiraceae widely exists in human gut [29] and is related to some severe diseases such as non-alcoholic fatty liver disease and inflammatory bowel diseases *et al* [30,31]. Since this important taxon is identified by both models, this indicates that both ZILI and GGM can explain part of the information encoded in the relative abundance and the ZILI model can serve as a competitive tool for the MIN selection.

The organization of this paper is as follows. In “Zero-inflated latent Ising model for MIN” section, the ZILI model is detailed. The related estimation procedures for ZILI are described in “MIN selection based on ZILI” section. Simulation studies are carried out in “Results from the simulated data” section. “Restults from the relative abundance of gut microbiota” section is devoted to compare ZILI and GGM through gut microbiome dataset. “Discussion” section concludes with a brief review about ZILI model.

## Method

### Zero-inflated latent Ising model for MIN

In this section, we introduce the zero-inflated latent Ising (ZILI) model for the microbial interaction network which provides an alternative way to handle the problem of unit-sum constraint and zero inflation. Suppose that there are *p* taxa in the microbiota of interest. For *j*th taxon (*j*=1,⋯,*p*), let *Z*_*j*_ denote its latent state variable which has the following multinomial distribution,

1 $$\begin{array}{@{}rcl@{}} P(Z_{j}=k)=p_{jk} \end{array} $$

for *k*=0,1,⋯,*K*_*j*_−1 with $\sum _{k=0}^{K_{j}-1}p_{jk}=1$, where *K*_*j*_ represents the number of the latent states for *j*th microbe (1≤*j*≤*p*). For example, there may be three states for *Z*_*j*_ corresponding to three different states of relative abundance, (high, medium, low). This assumption can be partly justified by the existing findings in literature [28]. The studies in [28] found that the composition of vaginal bacterial communities can be characterized by five states. The microbiota for a given subject can be classified into one of these five states. The state may be affected by the exogenous factors such as sexual activity, menstruation et al. In order to study the general relationship among the microbiota, Eq. (1) generalizes the results in [28] and assumes there are finite states for each microbe. For ease of exposition, in the following we assume that all *Z*_*j*_’s are *K*-level variables. The arguments can be generalized to the more general situation straightforwardly for which *K*_*j*_ may differ for different microbes. We pool all the *Z*_*j*_’s together and form the vector **Z**=(*Z*_1_,⋯,*Z*_*p*_) for which multiclass Ising model is employed to characterize its joint distribution,

2 $$\begin{array}{@{}rcl@{}} P(\mathbf{z})=c\exp \left\{\sum_{s=1}^{p} \phi_{s}(z_{s})+\sum_{s=1}^{p}\sum_{t=1}^{p} \phi_{st}(z_{s},z_{t})\right\}, \end{array} $$

where *ϕ*_*s*_ and *ϕ*_*st*_ are the potential functions associated with *s*th and *t*th microbes respectively. It should be noted that conventionally the variables in Ising model are dichotomous. The general cases of multiple values are usually referred as Potts model in literature [32]. However, in order to keep the notation consistent with recent studies, e.g., [33], with a little abuse of notation, we use the multiclass Ising model to refer to the Potts model. Our aim is to estimate the conditional relationship among *Z*_*j*_’s these potential functions can be parameterized as follows. For each 1≤*s*≤*p*, and *l*∈{0,⋯,*K*−1}, define *I*[*z*_*s*_=*l*]=1 if *z*_*s*_=*l* and 0 otherwise. Then we have

3 $$ \phi_{s}(z_{s})=\sum_{l\in \mathcal{A}}\boldsymbol{\theta}_{s;l}I[z_{s}=l]  $$

for *s*∈{1,2,⋯,*p*} and $\mathcal {A}=\{1,\cdots,K-1\}$ while

4 $$ \phi_{st}\left(z_{s},z_{t}\right)=\sum_{(l,h)\in \mathcal{B} }\theta_{st;lh}I\left[z_{s}=l;z_{t}=h\right]  $$

for (*s*,*t*)∈{1,⋯,*p*}^2^ and $\mathcal {B}=\mathcal {A}\times \mathcal {A}$. The unknown parameters in (3)-(4) include ***θ***={*θ*_*j*;*l*_,*θ*_*jt*;*lh*_*j*=1,⋯,*p*,*t*=1,⋯,*p*,*j*≠*t*,*l*=1,⋯,*K*−1,*h*=1,⋯,*K*−1}.

Based on (2)-(4), for 1≤*i*≤*n*, 1≤*j*≤*p*, we have the following equation hold [33,34],

5 $$\begin{array}{@{}rcl@{}} {p_{jl}\stackrel{\Delta}{=}\text{logit}}\left(P\left[Z_{ij}=l|\mathbf{Z}_{i(-j)}=\mathbf{z}_{i(-j)}\right]\right)= \theta_{j;l}+\sum_{t\neq j}\sum_{h=1}^{K-1}\theta_{jt;lh}I[z_{it}=h], \end{array} $$

where **Z**_*i*(−*j*)_=(*Z*_*i*1_,⋯,*Z*_*i*(*j*−1)_,*Z*_*i*(*j*+1)_,⋯,*Z*_*ip*_)^*T*^ with *Z*_*ij*_ the *i*th observation of *Z*_*j*_. From (5), it can be shown that *θ*_*j*;*l*_ is the log odds for event *Z*_*j*_=*l* given that the other *Z*_*t*_’s, *t*≠*j* are all zero. Similarly, *θ*_*jt*;*lh*_ is the log-odds ratio describing the association between events *Z*_*j*_=*l* and *Z*_*t*_=*h* given that all the other components of **Z** are fixed to zero. For more details about the interpretation of these quantities, see [33] and references there. Let ***θ***_*jt*_=(*θ*_*jt*;11_,⋯,*θ*_*jt*;1(*K*−1)_,*θ*_*jt*;(*K*−1)1_,⋯,*θ*_*jt*;(*K*−1)(*K*−1)_)^*T*^. Vector ***θ***_*jt*_ reflects the relationship between *Z*_*j*_ and *Z*_*t*_. If all the components of ***θ***_*jt*_ are zero, *Z*_*j*_ and *Z*_*t*_ turn out to be independent. If there exist nonzero components in ***θ***_*jt*_, then *Z*_*j*_ and *Z*_*t*_ are related. In other words, there is an edge connecting microbe *j* and *t* in the microbial interaction network.

**Fig. 1:**
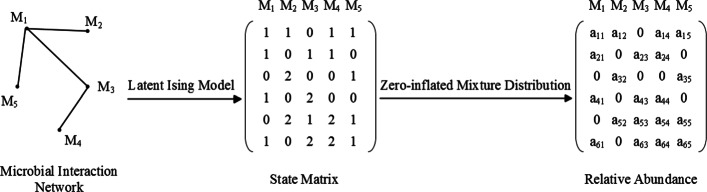
Diagram of data-generating process in ZILI model

We have assumed that the relationship among microbes can be characterized by the multiclass Ising model (2)-(4). The state variables *Z*_*j*_’s in Ising model, however, are latent and can not be observed directly. Instead, the observable quantities are the relative abundances of the microbes which ae denoted by *X*_*j*_’s here. For each *X*_*j*_, we assume its distribution can be characterized by a mixture distribution which relies on the realization of **Z**. Specifically, we have the following conditional distribution for *X*_*j*_ given *z*_*j*_=*l* for 1≤*l*≤*K*−1,

6 $$\begin{array}{@{}rcl@{}} f\left(x_{j}|z_{j}=l\right)= f_{jl}(x_{j}), \end{array} $$

where *f*_*jl*_ (1≤*l*≤*K*−1) can be any continuous distribution defined on [0,1]. When *z*_*j*_ is in state zero, i.e., *l*=0, we assume

$$f_{j0}(x)=\left\{ \begin{array}{ll} \pi_{j} &\quad \text{for}\ x=0\\ g_{j0}(x) & ~~~~\text{otherwise} \end{array}\right. $$

for some 0<*π*_*j*_<1. Here *g*_*j*0_ can be any continuous distribution defined on [0,1]. In other words, *f*_*j*0_(*x*) is a zero-inflated distribution. Let *μ*_*jl*_=*E*(*X*_*j*_|*Z*_*j*_=*l*). For *l*=0, *μ*_*jl*_ is understood as the expectation with respect to the density function *g*_*j*0_. Note if there are states, *l*≠*h* such that *μ*_*jl*_=*μ*_*jh*_, then it is impossible to identify state *l* from *h* based on absolute or relative abundance data. In order to ensure the model identifiability, without loss of generalization, we assume,

7 $$\begin{array}{@{}rcl@{}} \mu_{j0}<\mu_{j1}<\cdots<\mu_{j(K-1)} \end{array} $$

for 1≤*j*≤*p*. Given **X**=(*X*_1_,⋯,*X*_*p*_) and its *n* i.i.d observations, **X**_1_,⋯,**X**_*n*_, we aim to estimate the MIN through (1) ∼(7) which we call zero-inflated latent Ising model (ZILI). The data-generating process of ZILI is depicted in Fig. 1.

#### **Remarks 1**

(1) We have adopted a zero-inflated form for density function *f*_*j*0_ while continuous form for *f*_*jl*_ (1≤*l*≤*K*−1). In other words, the zero observations can only arise from *f*_*j*0_ which has the smallest mean relative abundance among *f*_*j*0_,⋯,*f*_*j*(*K*−1)_. This assumption serves to ensure the identifiability of ZILI model. In literature, the zero observations in microbiome data are usually classified into two categories by their nature [2,6]. In first category, the zero means the corresponding microbe physically does not exist in the subject, or true zero. In second category, the microbe does exist in the subject; nevertheless, for this sample, this microbe happens not to exist or be below the threshold of the testing procedure, i.e., false zero. So our assumption about *f*_*jl*_ for 0≤*l*≤*K*−1 means that both true and false zero’s can only come from *f*_*j*0_. Though there is possibility that there are zeros’ that do come from *f*_*jl*_(*l*≠0), we assume such probability is negligible compared with the former case, which we believe is a reasonable simplication to the real situations. (2) Conventional latent models, e.g state space model, typically assume the observed variables can be represented by a small number of latent variables and in this way the model dimensionality can be reduced. In ZILI, however, the latent variables are for the observations instead of the observed variables. Similar ideas have been used in factor analysis with the name R-type or Q-type factor analysis respectively [35].

### MIN selection based on ZILI

From Eq. (5), it can be seen that the selection of MIN is equivalent to the selection of the nonzero components of ***θ*** involved in ZILI model. In this section, we propose a two-step algorithm to select such nonzero components of ***θ*** based on **X**_1_,⋯,**X**_*n*_, the observations of relative abundance.

#### Step 1: state estimation

In this step, for each microbe, we aim to estimate the state *Z*_*j*_ (1≤*j*≤*p*) for each observation. For any given microbe, the proposed algorithm only involves its own observations. So for ease of exposition, we suppress the subscript *j* and use the generic notation (*Z*,*X*) to introduce the algorithm. The corresponding number of classes is denoted by *K*_*j*_=*K*.

With the observations of relative abundance, *X*_1_, *X*_2_, ⋯, *X*_*n*_, in hand, the estimation of *Z* is carried out through the following optimal classification of *X*_1_, *X*_2_, ⋯, *X*_*n*_. Without loss of generality, we assume that the observations have been ordered, i.e., *X*_1_≤*X*_2_≤⋯≤*X*_*n*_. For a given integer *k*≥2, let *b*(*n*,*K*) denote a classification scheme which classifies (*X*_1_,⋯,*X*_*n*_) into *k* classes. Such classification can be depicted by the following notations,

8 $$\begin{array}{@{}rcl@{}} G_{1}&=&\left\{X_{1}, X_{2}, \cdots,X_{i_{1}}\right\},\\ G_{2}&=&\left\{X_{i_{1}+1}, X_{i_{1}+2}, \cdots,X_{i_{2}}\right\},\\ \cdots&\cdots&\cdots\cdots\cdots\cdots,\\ G_{k}&=&\left\{X_{i_{K-1}+1},\cdots X_{n}\right\}. \end{array} $$

With notation *i*_0_=1,*i*_*k*_=*n*, we define the following loss function for *b*(*n*,*K*),

$$L[b(n,K)]=\sum_{h=0}^{K-1} D\left(i_{h}, i_{h+1}\right), $$

where

9 $$\begin{array}{@{}rcl@{}} D\left(i_{h}, i_{h+1}\right)=\sum_{i=i_{h}}^{i_{h+1}} \left(X_{i}-m_{h}\right)^{2},\\ m_{h}=\frac{1}{i_{h+1}-i_{h}+1}\sum_{i=i_{h}}^{i_{h+1}} X_{i}. \end{array} $$

We aim to find a classification scheme *b*(*n*,*K*) which can minimize loss function *L*[*b*(*n*,*K*)]. Such optimal classification scheme is denoted by *p*(*n*,*K*). It should be noted that other more complext forms of loss function *D*(·) are also possible, e.g., the absolute deviation based loss function, which is more robust for the data with outliers. However, given the popularity of squared loss function, we will focus on (9) and leave other possible forms for the future studies. We employ the following top-down dynamic programming algorithm to find *p*(*n*,*K*) [36]. Specifically, the algorithm involves the following recursive procedures,

10 $$\begin{array}{@{}rcl@{}} L[p(n,2)]&=&\min_{2\leq i\leq n}\left\{D(1,i-1)+D(i,n)\right\}, \end{array} $$

11 $$\begin{array}{@{}rcl@{}} L[p(n,K)]&=&\min_{K\leq i\leq n}\left\{L\left[p(i-1,K-1)\right]+D(i,n)\right\}. \end{array} $$

Based on (10)-(11), for given *K*, the algorithm can be implemented as follows. First, find *i*_*K*−1_ such that

12 $$\begin{array}{@{}rcl@{}} L[p(n,K)]=L\left[p(i_{K-1}-1,K-1)\right]+D(i_{K-1}, n). \end{array} $$

Based on *i*_*K*−1_, denote the *K*th class by *G*_*K*_={*i*_*K*−1_,*i*_*K*−1_+1,⋯,*n*}. In second step, find *i*_*K*−2_ such that

$$L\left[p\left(i_{K-1}-1,K-1\right)\right]=L\left[p\left(i_{K-2}-1,K-2\right)\right]+D\left(i_{K-2},i_{K}-1\right), $$

then we get the (*K*−1)th class *G*_*K*−1_={*i*_*K*−2_,*i*_*K*−2_+1,⋯,*i*_*K*−1_−1}. By the same fashion, all the classes *G*_1_,*G*_2_,⋯,*G*_*K*_ can be derived, which is the optimal solution *p*(*n*,*K*). Based on *p*(*n*,*K*), the estimate of *Z* for observations in class *G*_*k*_ is defined as $\hat {Z}=k-1$ for *k*=1,⋯,*K*.

The algorithm above assumes that *K*, the number of the classes, is known as a priori. In practice, *K* usually is unknown and has to be determined based on the data. Though several methods have been proposed in literature, such as likelihood ratio test in R package *mixtools* [37], or BIC method in package *sBIC* [38], these methods have poor performances when the data are zero-inflated. Instead, we propose the following criterion to select *K*. For a given upper bound, say, $\bar {K}$, and each *K* with $2\leq K\le \bar {K}$, the minimum loss *L*(*p*(*n*,*K*)) is calculated. Define *d*_*K*_=*L*(*p*(*n*,*K*+1))−*L*(*p*(*n*,*K*)) for $K=2,\cdots, \bar {K}-1$ and let $\bar {d}$ be the mean of *d*_*K*_’s. Then the first *K* with $d_{K}\leq \bar {d}$ will be selected as the class number. This criterion turns out to have a better performance than the methods mentioned above in the simulation studies in “Results from the simulated data” section. However, it should be noted, as suggested by one of the reviewers, that the choice of *K* may potentially have big impact on the final selected model. Consequently, in practice the robust way for the determination of *K* is to compare multiple methods, from which domain knowledge may be employed to choose the optimal one.

#### Step 2: network selection

Equation (5) shows that, after the logit transformation, the conditional probability *p*_*jl*_ defined in (5) is a linear function of ***θ***. Here the covariates are the indicator functions of events {*Z*_*it*_=*h*} (*t*≠*j*,*h*=1,⋯,*K*−1). Based on this observation, the neighborhood method is proposed in [33] to select the nonzero components in ***θ*** for dichotomous Ising model. In this paper the same method will be employed for the MIN selection. Specifically, for *j*th microbe, let $\boldsymbol {\theta }_{j}=\left (\boldsymbol {\theta }_{j1}^{T},\cdots, \boldsymbol {\theta }_{j(j-1)}^{T}, \boldsymbol {\theta }_{j(j+1)}^{T}, \cdots,\boldsymbol {{\theta }}_{jp}^{T}\right)^{T}$ where ***θ***_*jt*_ is defined in “Method” section. Based on the Eq. (5), we consider the following penalized group logistic regression problem,

13 $$\begin{array}{@{}rcl@{}} \hat{\boldsymbol{\theta}}_{j}={\arg\min}_{\boldsymbol{\theta}_{j}}\left\{-l\left(\boldsymbol{\theta}_{j}|\hat{\mathbf{Z}}_{(-j)}\right)+\lambda\sum_{t\neq j}\sqrt{m_{jt}}\|\boldsymbol{\theta}_{jt}\|_{2}\right\}, \end{array} $$

where $l(\boldsymbol {\theta }_{j}|\hat {\mathbf {Z}}_{(-j)})=\sum _{i=1}^{n} \left \{I(Z_{ij}=l)\log p_{jl}+(1-I(Z_{ij}=l))\log (1-p_{jl})\right \}$ with *p*_*jl*_ being defined in eq. (5), *λ* is the tuning parameter, *m*_*jt*_ is the length of vector ***θ***_*jt*_ and ∥·∥_2_ is the Euclidean norm. The form of $\sqrt {m_{jt}}$ aims to account for the varying group size of ***θ***_*jt*_ [39]. Such form of penalty in (13) tends to shrink the components in same group ***θ***_*jt*_ to zero simultaneously. For given *λ*, the coordinate decent algorithm [40,41] is employed to solve (13). As for the selection of *λ*, extended BIC proposed in [42] is adopted which favors sparser model compared with the standard BIC. The minimization problem in (13) is solved for each *Z*_*j*_ (1≤*j*≤*p*). With the final estimate $ \hat {\boldsymbol {\theta }}$ in hand, we define an edge between *Z*_*j*_ and *Z*_*t*_ if there exists at least one nonzero component in either $\hat {\boldsymbol {\theta }}_{jt}$ or $\hat {\boldsymbol {\theta }}_{tj}$. An alternative way to define an edge requires there exists at least one nonzero component in both $\hat {\boldsymbol {\theta }}_{jt}$ and $\hat {\boldsymbol {\theta }}_{tj}$. It turns out these two strategies are asymptotically equivalent [33,43] and so we just employ the former one to select the MIN in the numerical studies. The magnitude of the components of $\hat {\boldsymbol {\theta }}_{jt}$ plays no role in the determination of the edges [33,44].

**Fig. 2:**
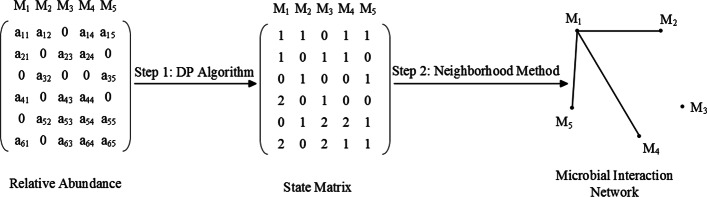
Procedures of two-step algorithm for ZILI model

In the above, the proposed algorithm estimates the interaction network by separately solving *p* conditional penalized maximum likelihood estimation problems. Alternatively, we can form a joint conditional likelihood function for ***θ*** and estimate the network through the penalized version of the joint conditional likelihood function; however, this approach is not computationally as stable as (13) [44]. We therefore put the focus on the individual regression method (13). Figure 2 illustrates the workflow of the two-step algorithm through a toy MIN.

##### **Remarks 2**

For the two-step algorithm proposed above, it is expected that the selection of MIN will be improved if we can improve the state estimates $\hat {Z}_{ij}$’s; however, the misclassification is inevitable in two-step algorithm which will adversely impact the final network selection. In “Results from the simulated data” section, we investigate how the misclassification impacts the MIN selection through simulation studies.

## Results

### Results from the simulated data

In this section, we investigate the performance of the two-step algorithm when ZILI is the underlying data-generating model. As a comparison, the popular Gaussian graphical model (GGM) and dichotomous Ising model (DIS) will also be fitted using the same dataset. Here DIS is constructed by transforming the relative abundance into 0 or 1 according to whether it is less than the median. The same algorithm in “Step 2: network selection” section will be employed to estimate the structure of this dichotomous Ising model.

Specifically, assume that there are *p* microbes with state variables **Z**=(*Z*_1_,⋯,*Z*_*p*_). Each realization of *Z*_*j*_ (*j*=1,⋯,*p*) takes value from the set {0,1,2}. The conditional distribution of *Z*_*j*_ (*j*≠1) given all the other components of **Z** only depends on microbe *Z*_*j*−1_. As for microbe 1, the distribution of *Z*_1_ depends on microbe *Z*_*p*_. For such a model, the nonzero parameters involved in Eq. (5) include (*θ*_*j*;1_,*θ*_*j*;2_,*θ*_*j*(*j*−1);11_,*θ*_*j*(*j*−1);12_,*θ*_*j*(*j*−1);21_,*θ*_*j*(*j*−1);22_) which are assumed to be same for all *j*’s. For each replication, these parameters are sampled from the multivariate normal distribution *N*_6_(*μ*,*Σ*) with *μ* =(−1,3,−0.8,2,−3,−4)^*T*^ and *Σ*=diag(0.1^2^,0.3^2^,0.08^2^,0.2^2^,0.3^2^,0.4^2^).

Given the Ising model above, the Gibbs sampler is employed to generate the samples of **Z**. Specifically, first a *p*-dimensional vector is generated where the states for each *Z*_*j*_ are independently sampled from the set {0,1,2} with equal probability 1/3. Then given all *Z*_*t*_, (*t*≠*j*), the state of *Z*_*j*_ is updated based on Eq. (5). By the same fashion, the states of all the other *Z*_*j*_ can be updated recursively. We run this process 200 times and the final state of **Z** will be deemed a qualified representative of the underlying Ising model. Based on the samples of **Z**, the samples of absolute abundance **X**=(*X*_1_,⋯,*X*_*p*_) are generated according to *X*_*j*_|*Z*_*j*_=*z*∼N(*μ*_*z*_,*σ*^2^) with *μ*_0_=10,*μ*_1_=15,*μ*_2_=20 and a given *σ*^2^. Pooling all the samples of **X** together leaves us a *n*×*p* matrix which represents *n* absolute abundance observations for *p* microbes. For each column, the absolute abundances which are less than a given percentile with rank *u* are replaced by zero. Here *u* is sampled from uniform distribution *U*[0,*τ*] for a given 0<*τ*<1. For each row in this zero-inflated matrix, we then transform the absolute abundances to relative abundances by dividing each entry by the corresponding row sum. Figure 2 shows the diagram for the data-generating process.

To compare the performances of different models, two criteria, true positive rate (TPR) and false positive rate (FPR) will be used which are defined respectively as,

14 $$\begin{array}{@{}rcl@{}} \text{TPR}&=&\frac{\#\{\text{identified\ true\ edges}\}}{\#\{\text{all\ true\ edges}\}}, \end{array} $$

15 $$\begin{array}{@{}rcl@{}} \text{FPR}&=&\frac{\#\{\text{falsely\ identified \ edges}\}}{\#\{\text{all \ none\ \ edges}\}}. \end{array} $$

An ideal algorithm should have a relatively high TPR and low FPR. There are multiple factors that can influence the performance of the algorithm, which include the variance *σ*^2^, the sample size *n*, and the zero proportion *z*. For three choices *σ*, two choices of *n* and three choices of *τ*, Table 1 lists the results of TPR and FPR for ZILI, DIS and GGM respectively. Here the number of the microbes is set to be *p*=60 and the number of replication is 100. Note for GGM, there are different estimation methods available such as graphical lasso [45], or neighborhood method [43] et al. Here in order to facilitate the comparison with ZILI and DIS, we adopt the neighborhood method of [43]. The same model selection criterion extended BIC is used in all cases. It can be seen from Table 1 that for all the cases considered, the proposed two-step algorithm does can select the network structure effectively while both GGM and DIS have low TPR and can not properly select the true edges. On the other hand, all the three factors considered, i.e., variance, sample size and zero proportion have significant impact on the performances of two-step algorithm. Two-step algorithm has the best performance with the small *σ*^2^, *τ* and large *n* which is in accordance with our expectation. In particular, a large *σ*^2^ will lead to a high misclassification rate for the state estimation which in turn results in a poor network selection, i.e., low TPR and high FPR.

**Table 1:** Comparison of ZILI, DIS and GGM based on simulated data

				**ZILI**			**DIS**			**GGM**	
*τ*	*σ*	*n*		TPR	FPR		TPR	FPR		TPR	FPR
10	0.5	60		0.8322	0.0110		0.0115	0.0008		0.1940	0.0347
		120		0.9650	0.0024		0.0173	0.0006		0.0721	0.0058
	1	60		0.7945	0.0123		0.0106	0.0007		0.0145	0.0059
		120		0.9615	0.0043		0.0163	0.0005		0.1683	0.0051
	2	60		0.2688	0.0256		0.0115	0.0010		0.0123	0.0061
		120		0.5041	0.0187		0.0096	0.0003		0.0368	0.0046
40	0.5	60		0.7775	0.0134		0.0106	0.0009		0.1961	0.0274
		120		0.9445	0.0059		0.0180	0.0005		0.1923	0.0056
	1	60		0.7260	0.0139		0.0163	0.0007		0.1895	0.0276
		120		0.9353	0.0067		0.0120	0.0003		0.1821	0.0057
	2	60		0.2085	0.0247		0.016	0.0013		0.1328	0.030
		120		0.4043	0.0194		0.0115	0.0004		0.0991	0.0055
80	0.5	60		0.4443	0.0217		0.0720	0.0086		0.2346	0.0465
		120		0.6090	0.0148		0.1115	0.0071		0.2248	0.0144
	1	60		0.4088	0.0214		0.0681	0.0085		0.2321	0.0477
		120		0.6020	0.0149		0.1138	0.0071		0.2196	0.0146
	2	60		0.1538	0.0247		0.0493	0.0084		0.1851	0.0514
		120		0.2820	0.0207		0.0938	0.0065		0.1620	0.0142

### Restults from the relative abundance of gut microbiota

In this section, ZILI is employed to investigate the conditional association among the microbes in the infant stool samples from New Hampshire Birth Cohort Study (NHBCS), a cohort of mother-infant pairs in New Hampshire. For this dataset, stool samples were collected from infants at six weeks and twelve months of age, who were followed in the NHBCS. The stool samples were characterized by 16S rRNA sequencing. The R software package *DADA21* was used to infer the abundance of amplicon sequence variants in each sequenced sample [46]. Taxonomy at the family level was obtained by classifying the sequences against the reference training dataset from the GreenGenes Database Consortium (Version 13.8). There were 398 six-week and 316 twelve-month samples with varying abundances across 134 taxonomic families.

For each taxon, if the proportion of nonzero observations is less than 1%, then the number of classes is set to be *K*_*j*_=2 and the observations are classified according to whether it is zero or not. Otherwise, the upper bound of *K*_*j*_ is set to be $\bar {K}= 6$. Then we follow the two-step algorithm to select the network. In order to gain insights from the difference between ZILI and GGM, the networks based on GGM have also been selected using the neighborhood method. In light of the severe zero inflation in the dataset, it is inappropriate to assume the GGM for the whole dataset. To alleviate the problem of zero inflation, we choose to use the subsets of this dataset to construct the GGM networks. Specifically, for each *s*=10*%*,20*%*,⋯,80*%*, we extract the corresponding subset from the original dataset which only includes the microbes whose proportions of nonzero observations are greater than *s*. For each of these subsets, GGM is fitted using the neighborhood method. The ZILI network involves 134 microbe taxa while the eight GGM networks only involves eight subsets of these 134 taxa. So in order to compare the ZILI network with the eight GGM networks, we extract the subnetworks from ZILI networks for each *s*. For each of the extracted network, we then compare it with the corresponding GGM network in terms of their connectivity and the results are listed in Table 2.

**Table 2:** Comparison of microbial interaction networks selected by GGM and ZILI

		**ZILI**				
		**(0,0)**	**(1,0)**	**(0,1)**	**(1,1)**	***p*-value**
	10%	589	58	6	13	0.0000
	20%	357	37	3	9	0.0000
	30%	182	38	2	9	0.0000
GGM	40%	137	25	2	7	0.0000
	50%	89	23	2	6	0.0022
	60%	55	17	0	6	0.0005
	70%	46	15	0	5	0.0252
	80%	19	6	0	3	0.0445

The data are the relative abundances of microbiota in infant gut from NHBCS

In Table 2, each row corresponds to a pair of ZILI and GGM networks. For two microbes, (0,0) represents there is no edge connecting them in both ZILI and GGM network; (0,1) represents there is an edge in ZILI network while no edge in GGM network; (1,0) represents there is an edge in GGM network while no edge in Ising network; (1,1) represents there is an edge connecting them in both ZILI and GGM network. The columns 3-6 in Table 2 list the numbers of the edges falling into these four categories respectively. The relationship of ZILI and GGM is our primary interest. To this end, the *χ*^2^ test for the independence of ZILI and GGM is carried out and the corresponding adjusted *p*-value’s are listed in the last column of Table 2. Note the *p*-value here is based on the estimated networks rather than the relative abundance. So we call them conditional *p*-value. These *p*-value’s suggest that the networks of ZILI and GGM are closely related, even though ZILI and GGM are based on entirely different assumptions about how the data are generated. A more detailed inspection reveals that most of the edges selected by ZILI are also selected by GGM and GGM selects far more edges than ZILI. In other words, ZILI is more conservative than GGM in terms of edge selection.

**Fig. 3:**
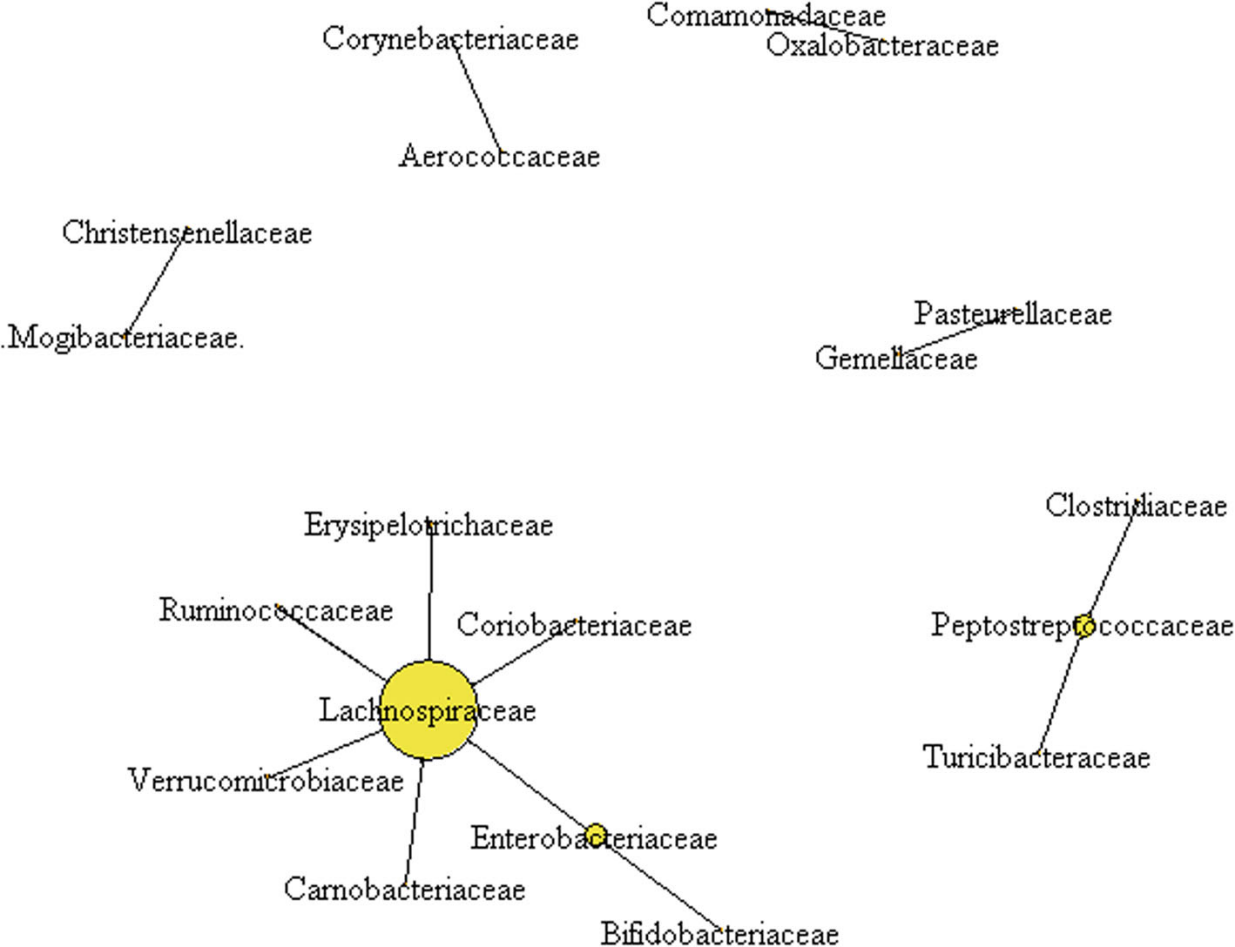
The overlappted subnetwork in GGM network and ZILI nework

The ZILI network and all the GGM networks corresponding to the threshold *s*=10*%*,20*%*,⋯,80*%* are available in the supplementary materials. Figure 3 presents the subnetwork that is shared by the ZILI networks and GGM network corresponding to *s*=10*%*. From Fig. 3, it can be seen that Lachnospiraceae is selected as hub taxon by both ZILI and GGM. It has been discovered in literature that R. gnavus, one of the members in Lachnospiraceae family, has high frequency in infant gut [29]. Lachnospiraceae has close connections with severe human diseases, such as inflammatory bowel diseases (IBD) [30], non-alcoholic fatty liver disease [31]. The R. gnavus ATCC 29149 strain possesses the complete Nan cluster involved in sialic acid metabolism for the production of an intramolecular trans-sialidase [47]. It has also been demonstrated recently that R. gnavus produces iso-bile acids. The iso-bile acids detoxification pathway influences the growth of one of the predominant genera in the human gut, i.e., the Bacteroides [48]. In summary, Lachnospiraceae plays an active role in human metabolism which in turn impacts the growth of the other taxa in the gut microbiota. In this respect, it is not surprising to find its wide connections with other members of the microbiota.

## Discussion

The prosperous microbiome datasets have led us to a new level of biological researches. Nevertheless, how to gain scientific insight from these complex datasets through novel statistical methods remains a big challenge for researchers. In light of the difficulties in MIN selection, we propose a novel zero-inflated latent Ising model (ZILI) to this problem. In ZILI, the relative abundances of microbiota are assumed to follow a mixture distribution which relies on the realization of a latent Ising model. Through simulation studies, it is shown that under given scenarios, the proposed two-step algorithm for the inference of ZILI can select the true network structure effectively while Gaussian graphical model and dichotomous Ising model have little power to recover the network structure. For a microbiome dataset from New Hampshire Birth Cohort Study, it is shown that ZILI is more conservative compared with Gaussian graphical model. Among the edges shared by these networks, a hub taxon is selected which has close connections with human metabolism. These findings indicate that ZILI can serve as an competitive model to estimate the microbial interaction network. On the other hand, we only consider the problem of model selection in this paper. In order to gain more insights about the conditional correlation beween microbes, quantitative characteristics like parameter estimates should be taken into consideration which will be studied in our future studies.

## Supplementary information

**Additional file 1** The file *Networks*.*pdf* includes the gut microbial interaction networks selected by ZILI model and Gaussian graphical model with thresholds, 10%, 20%, 30%, 40%, 50%, 60%, 70%, 80% respectively. These networks are used in Table 2 to investigate the relationship between ZILI and GGM.

## Data Availability

The relative abundance datasets used for this work are available upon request from the corresponding author. The programs used to implement the algorithms in this paper is written with R langurage and can be freely downloaded at github website, https://github.com/hoenlab/Ising.
