# Supplementary material for: Identification of microbial interaction network: zero-inflated latent Ising model based approach
Source: BioData Min. 2020 Oct 7;13:16. doi: 10.1186/s13040-020-00226-7 (PMC7542390; doi:10.1186/s13040-020-00226-7)
Supplement: Supplementary file 1 — Additional file 1 The file Networks.pdf includes the gut microbial interaction networks selected by ZILI model and Gaussian graphical model with thresholds, 10%, 20%, 30%, 40%, 50%, 60%, 70%, 80% respectively. These networks are used in Table 2 to investigate the relationship between ZILI and GGM. [file 13040_2020_226_MOESM1_ESM.pdf]

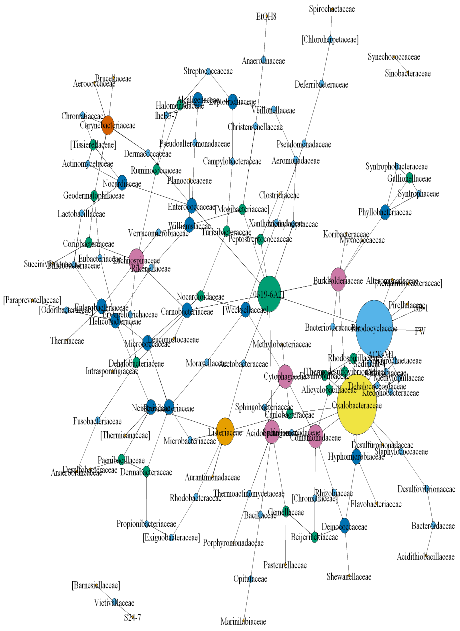

Figure 1: The network selected by ZILI

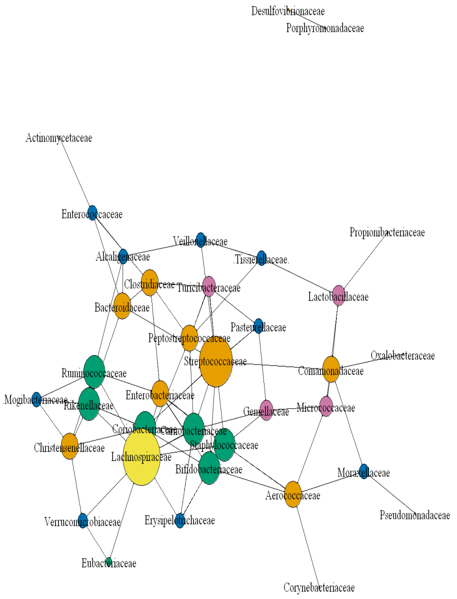

Figure 2: GGM network for threshold $s = 0.1$

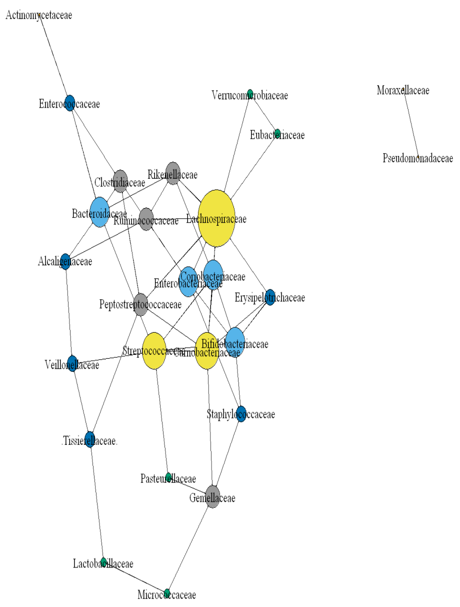

Figure 3: GGM network for threshold $s = 0.2$

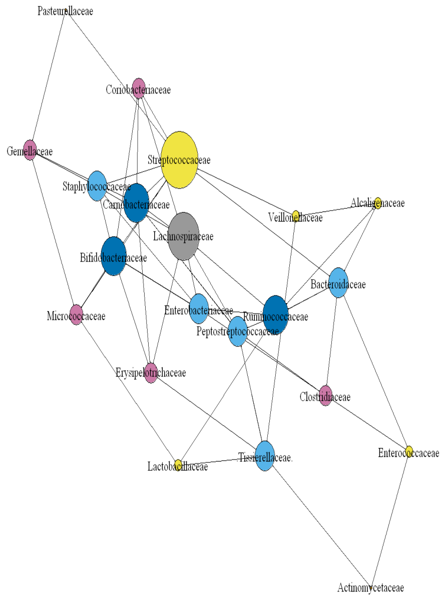

Figure 4: GGM network for threshold $s = 0.3$

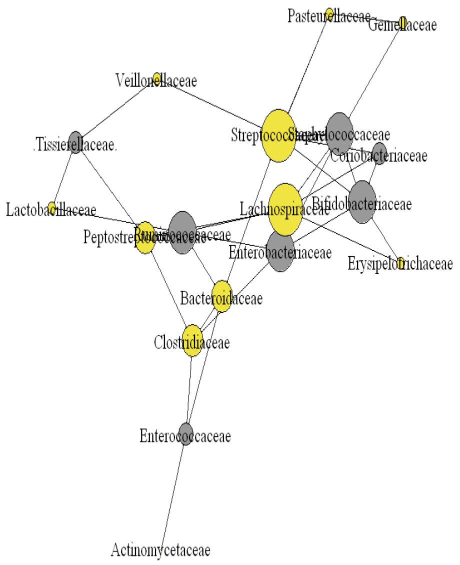

Figure 5: GGM network for threshold $s = 0.4$

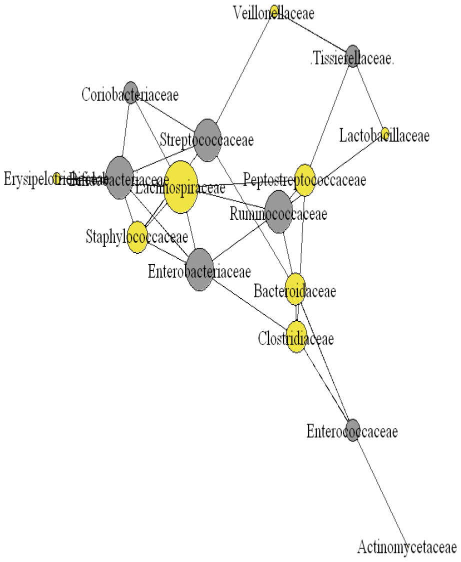

Figure 6: GGM network for threshold $s = 0.5$

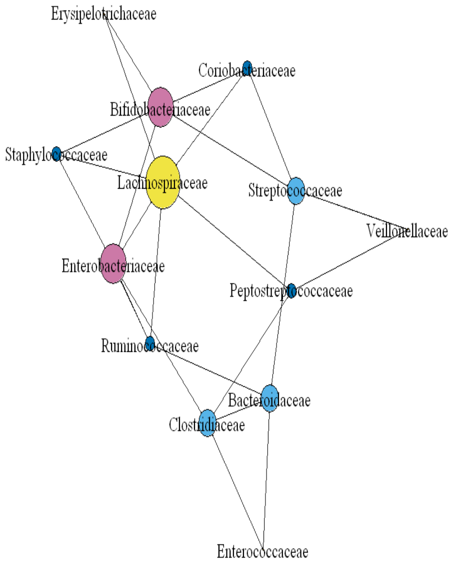

Figure 7: GGM network for threshold $s = 0.6$

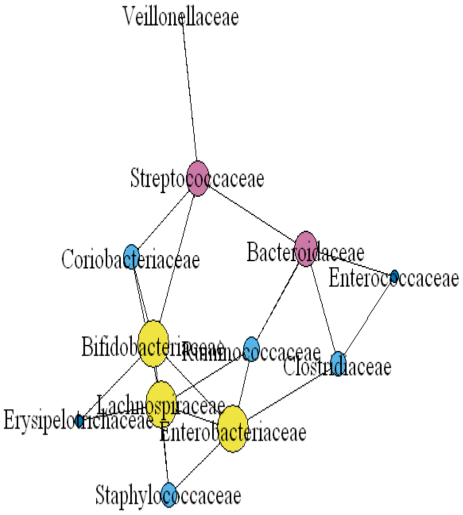

Figure 8: GGM network for threshold $s = 0.7$

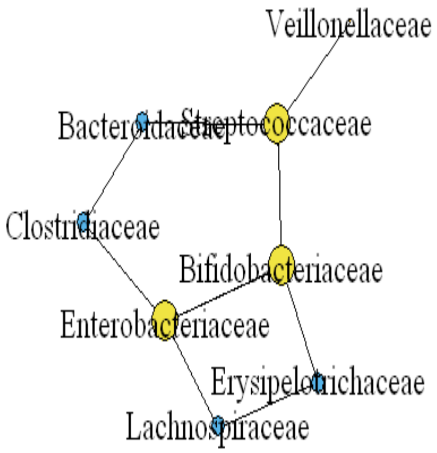

Figure 9: GGM network for threshold $s = 0.8$
